# The Female Sex Work Industry in a District of India in the Context of HIV Prevention

**DOI:** 10.1155/2012/371482

**Published:** 2012-12-30

**Authors:** Raluca Buzdugan, Shiva S. Halli, Jyoti M. Hiremath, Krishnamurthy Jayanna, T. Raghavendra, Stephen Moses, James Blanchard, Graham Scambler, Frances Cowan

**Affiliations:** ^1^Department of Infection and Population Health, University College London, London, UK; ^2^School of Public Health, University of California Berkeley, Berkeley, CA 94704, USA; ^3^Department of Community Health Sciences, University of Manitoba, Winnipeg, Canada; ^4^Karnataka Health Promotion Trust, Bangalore, India; ^5^St. John's Research Institute, St. John's Medical College, Bangalore, India

## Abstract

HIV prevalence in India remains high among female sex workers. This paper presents the main findings of a qualitative study of the modes of operation of female sex work in Belgaum district, Karnataka, India, incorporating fifty interviews with sex workers. Thirteen sex work settings (distinguished by sex workers' main places of solicitation and sex) are identified. In addition to previously documented brothel, lodge, street, *dhaba* (highway restaurant), and highway-based sex workers, under-researched or newly emerging sex worker categories are identified, including phone-based sex workers, parlour girls, and agricultural workers. Women working in brothels, lodges, *dhabas*, and on highways describe factors that put them at high HIV risk. Of these, *dhaba* and highway-based sex workers are poorly covered by existing interventions. The paper examines the HIV-related vulnerability factors specific to each sex work setting. The modes of operation and HIV-vulnerabilities of sex work settings identified in this paper have important implications for the local programme.

## 1. Introduction

Approximately 2.4 million people are living with HIV in India [1]. Most HIV transmission is heterosexual (particularly in the south) [2], and female sex workers (henceforth “sex workers”) are particularly vulnerable to HIV infection. Overall, 5.1% of sex workers are HIV infected nationally, with higher prevalence in some states or districts (e.g., Maharashtra—17.9%) [3, 4]. 

Indian sex workers work in various settings (e.g., brothels, lodges, and homes) [5]. Researchers and/or programmers have developed typologies to classify sex workers based on their work environment. These typologies are typically used in HIV programming design. For instance, India's National AIDS Control Organization (NACO) recommends that programmers take into account the sex work typology when designing sex worker interventions, conducting mapping exercises and outreach, and deciding outreach strategies [6]. 

Based on a review of sex work typologies in India, we showed that the most comprehensive national typology is the one proposed by NACO in 2007 [7]. This typology is based on sex workers' main place of solicitation, distinguishing between brothel-based, lodge-based, street-based, *dhaba*-based (i.e., restaurant located on highway), home-based, and highway-based sex workers [6]. The review also discussed three other documented sex work settings: parlour girls, agricultural workers involved in sex work, and phone-based sex work. 

Using data from Integrated Biological and Behavioural Assessment (IBBA) among Karnataka sex workers, we examined the extent to which the NACO typology predicts HIV risk. We showed that expanding the NACO typology to take account of both *place of solicitation* and *place of sex* better captures the HIV risk variation experienced by women working in different settings [8]. This extended typology distinguishes between women who solicit and have sex in brothels (brothel-to-brothel); women who solicit and have sex in their homes (home-to-home); and women who solicit in public places but have sex in various locations (street-to-home, street-to-rented-room, street-to-lodge, street-to-street). Street-to-lodge and brothel-to-brothel sex workers had the highest rates of HIV (30% and 34%, resp.) and of sexually transmitted infections (STI) (27% and 13%) and reported the highest client volume.

Following the analysis of the IBBA data, we conducted a qualitative study among women practicing sex work in various settings in Belgaum district, Karnataka state, to better understand the vulnerabilities faced by women practicing in particular settings. 

## 2. Methods

The study was conducted in March-April 2009 in Belgaum, which is one of the districts where the IBBA was conducted; this allowed us to explore the qualitative data in the context of the detailed information acquired through the quantitative survey. Of the five IBBA districts we attempted to select the district that was “average” but that also had a wide range of sex work settings. Among the five Karnataka districts that took part in the IBBA, Belgaum has the median rank on the human development index (HDI). (Out of the 27 districts in Karnataka, the ranks of the IBBA districts are as follows: Bangalore Urban 1, Shimoga 5, Belgaum 8, Mysore 14, and Bellary 18.) Moreover, Belgaum has a vibrant diverse sex work industry. 

The study aimed to examine HIV-related vulnerabilities across “all” sex worker categories, including those not represented in previous IBBA surveys. Based on discussions with programmers from the local NGOs, Karnataka Health Promotion Trust (KHPT) and Belgaum Integrated Rural Development Society (BIRDS), thirteen sex work settings (distinguished largely but not wholly by place of solicitation and place of sex) were identified: brothel, lodge, street-to-brothel, street-to-lodge, street-to-street, street-to-rented-room, street-to-home, home, phone-network, parlour, *dhaba*, highway and agricultural workers. An attempt was made to select four participants from each type of context, resulting in a total of fifty in-depth interviews with sex workers (Exceptions: 2 interviews with parlour girls, 3 with phone-based and home-based sex workers, and 5 with brothel-to-brothel and street-to-lodge sex workers.). The selection of participants was purposive to reflect these categories (judgment-based) [9].

Participants were recruited with the help of peer educators and other BIRDS staff, or by using the researchers' contacts in the sex work industry. The peer educators told women in the community about the study and asked for volunteers. The women willing to participate were introduced to the interviewer. The semistructured interviews took place at BIRDS drop-in-centres, women's residences, restaurants, and other public places, as long as the place allowed the interviewer to discuss with the participant in a quiet enabling environment. They were conducted by one of the authors in *Kannada* (local language), were audio-taped and translated. The interviewees' names or contact information were not recorded in any audio or paper-based study materials, thus ensuring anonymity of the data. While most participants were interviewed in one session, a second interviewing session was conducted if the first interview lacked information about a certain topic (following translation into English); while the identity of the participants was never recorded, the second session was possibly given the number of participants and the short period of time between the interview and its translation. Informed consent was obtained from all participants. The recordings of the interviews were saved on password-protected computers only accessible by the person analyzing the data and the translator. The study was approved by the ethics review boards of St. John's Medical College and Hospital, Bangalore, India and University College London, London, UK. 

Content analysis was conducted using Atlas.ti 5.0 and focused on the thematic areas explored in the interviews. Given that the objective of the study was to better understand the mode of operation and vulnerability factors of previously documented sex worker types [7, 8], the categories examined in the study were set prior to conducting the interviews. An ethnographic study aiming to develop a sex work typology might have decided the categories of sex workers using a grounded approach, rather than *a priori*, which may have required multiple interviewing sessions with each study participant.

After the English translation was finalized, the interviews were reread and coded in Atlas.ti according to the themes outlined below. Within each of these themes, the code list was developed while reading the interviews and was used to further code the interviews. The interviews were analysed by examining the coded text for each sex work setting (each code and theme at a time). 

In order to understand the mode of operation of each sex work context, the following thematic areas were examined: the place and mode of solicitation of clients, the place where they were entertained, the network operators, and the women's level of autonomy or agency. In order to facilitate an assessment of the HIV-related vulnerabilities of women working in different contexts, the following thematic areas were explored: the presence of network operators, the autonomy enjoyed by the women, the time spent per client, the experience of violence and harassment from various types of perpetrators, the main types of clients and their characteristics, the alcohol consumption of the women and their clients, the place and circumstances of condom negotiation, the exposure to the HIV prevention programme, the availability of condoms, and the women's condom negotiation skills. 

## 3. Results

### 3.1. Brothel

In Belgaum, brothels are houses where sex work takes place and are located in specific areas known for their sex work activities. While most clients are entertained in the brothels, regular clients are sometimes allowed to take the women to lodges (“If we know them, then we go with them… to the lodge”). The place of sex is decided by the brothel madam (*gharwali*), who exercises considerable control over the services on offer: she decides the rates, the “percentage” charged (usually 50%) (“If you sit in her house you pay half”), the number, and type of clients (“Whoever she sends we have to do, good people, bad people”). While the *gharwali*'s power over the women is indisputable, there is great variation in the way women are treated (“We both need each other. She has been of some help to us… She is like a parent to us. But some are like ‘You are only responsible for whatever happens'”). 

Women may have the option to leave the brothel depending on the circumstances of their recruitment, the “agreement” with the *gharwali,* and whether they owe her money. Some participants reported having been trafficked into brothels; while not specifically examined in this study, the vulnerability associated with trafficking has been widely documented and likely resulted in additional vulnerability among our participants [10, 11]. In general, if a loan stands against a particular sex worker (involving a person who trafficked her, her family, or the sex worker herself), she needs to remain in the brothel until the money is paid. Theoretically, if a sex worker does not owe the *gharwali* any money, she is free to leave the brothel at anytime. However, in practice, women tend to accrue considerable financial responsibilities, which make them take out loans on a more or less continuous basis (“If there is money that we need to repay her then we cannot go, but if we do not owe her any money then we can say “If not you, another *gharwali.*” True, we feel like that, but they do not let you go out. We have to have enough to pay her, is not it?”). 

Brothel sex workers typically have a relatively high client volume (up to 10 clients daily). The sex workers report that the *gharwali*'s attitude towards business and safe sex strongly affects the extent of the risks they face. Sex workers have little freedom to choose their clients, which can affect the consistency of condom use (“If we sent any client back she used to get angry, so we did not use [condoms] regularly”). They reported that while *gharwalis* are sometimes perpetrators of violence, but may also be protectors against other perpetrators that is, *gundas* (street thugs) (“[gundas] have to be given 50, 100 [Rupees] every week”), and the police. However, regardless of previous agreements between police and *gharwalis* (guaranteeing women's safety), the police can still be pressurized into closing brothels, for example due to negative media coverage, as happened in Belgaum in 2009 (“They showed on TV about our women… and it was a big problem and many of us had to leave”). 

Brothel sex workers tend to have clients from low-paid occupations, who are less inclined to use condoms [12]. Alcohol has been shown to increase sex workers' vulnerability [13–15]. Some of our brothel-based participants reported drinking to forget their problems (“Because of the tension, thinking about children, after coming here… I used to drink”). Many clients come to the brothel drunk or drink in the brothel (“Those who come for one time they used to drink and come, but those who stay for the night used to bring and drink”). 

Provided the *gharwali* cooperates with programme staff, brothel sex workers are easily accessible for outreach activities, because the location of the brothels is known and the women stay in the brothel on a relatively permanent basis. The women visit the drop-in-centre weekly (“We come to the office on Wednesdays”). 

### 3.2. Lodge

Some women solicit clients and have sex in lodges (small hotels). The location of lodges is known by regular clients (“Clients think ‘Why go searching for them?' They will be available in the lodge and they come there directly”). Most women come to their lodge daily, stay from morning until evening, with some also staying overnight. In other places (e.g., Mangalore, Karnataka), women reside in the lodge on a continuous basis, similar to brothel sex workers [16]. 

Lodge-based sex work is organized and involves a few network operators (“Room boys will be there, manager will be there to take rent, the owner will not be there”). The lodge provides the women with clients, decides the price for sex, and usually takes 50% of the fee (“200 Rupees will be charged, 100 they keep as room rent and 100 for us”). 

Unlike brothels, where *gharwalis* try to attract women to stay for many years (“It's like *gharwalis* want us to stay here only forever”), in lodges there is a high turnover of sex workers (“They will sleep with us once or twice, but why they will sleep with us always? They will say ‘These are not new'”). While lodge sex workers usually have the freedom to change lodges and/or move to another setting, usually the manager asks the sex worker to leave (“When new younger girls come, we have to leave, is not it?”). 

Lodge sex workers have a relatively high client volume (“10, 15 or some days just 5 [clients]”). The rates charged are twice those in brothels, allowing sex workers to make more money. While some women claim they can refuse clients, others explain that refusal equates to having to leave the lodge (“Whatever client comes, we have to do. They will say “A client is there, go do”, they threaten us, they won't allow us inside again otherwise”). There are a limited number of lodges in any locality (“This [lodge] is the only one that is open”), and because one can earn a good income in lodges there are always more interested women than places available. Hence, the balance of power is in favour of the owners/managers. Condom use in lodges likely depends on managers' attitudes, in that women are more likely to report condom use if they also report that their lodge managers are in favour of condom use and safe sex (“He [the lodge manager] says ‘use condom and do' but if he [the client] says it's not fun, then the manager says ‘Take your money and go, we get 100 other clients'”). 

Interviewees did not mention experiencing violence from managers or *gundas* (“there is no *gunda* problem in the lodge”). Despite agreements with the police (“They [the police] take ‘entry' [bribe]”), lodges are frequently raided, discouraging some women from working there (“In the lodge there is always fear about police raid”). While women are protected from violence from clients by the manager, some interviewees mentioned that violence occurs on a daily basis “if she does not sleep properly, if it does not open”. Most clients are “regulars,” and usually have low-paid occupations. Rooms are allocated to sex workers on rotation. The woman has 10 to 20 minutes to entertain the client (“We have to do and finish in ten minutes”); this time pressure can impact condom use. 

HIV programmes provide condoms (“These people bring… free of charge”), but sometimes sex workers need to buy condoms from the manager (“If we do not buy they say ‘Do not come here.'”). Programme staff usually know the lodges where sex work occurs. However, as with brothels, their access to women depends on the willingness of the owners/managers to cooperate. The women have little time to talk to peer educators and consequently tend not to visit drop-in-centres and interact with other sex workers (“Why should I go and sit there [drop-in-centre] leaving my *dhanda* [sex work]?”). 

### 3.3. Street

One of the most common sex work types in South India involves soliciting in public places, for example, bus stands, railway stations, markets, parks, and streets [2]. In Belgaum, street-based sex workers take their clients to brothels, lodges, public places, rented rooms, or their own homes. While autonomous, street-based sex workers will occasionally get clients through agents for example, auto-rickshaw drivers. 

Sex workers soliciting in public places share certain vulnerabilities *independent of where sex occurs*. While theoretically they can be selective about the type of clients they entertain, in practice their autonomy is limited by the fierce competition between women, due to limited availability of clients, and the low rates charged per client (“How can we refuse? We need to fill our stomach, is not it?”). Because they solicit clients in public places, they are highly visible and experience harassment and violence from *gundas* and the police (“They [*gundas*] pester, they ask for money”; “He [policeman] will sleep for free and go and some will say there will be a raid tomorrow and ask for money. We have to go [have sex with them]”). They report that most of their clients have low-paid occupations and are disinclined to use condoms. Regarding outreach, street-based sex workers are easy to identify and follow up and tend to visit the drop-in-centres regularly (“There is a place to sit. Where to sit outside? We can rest here, wash our face, freshen up and go”). In addition to vulnerability factors associated with street solicitation, there are factors specific to the place of sex, which are discussed in the next subsections. 

#### 3.3.1. Street-to-Brothel

Unlike those who work in brothels, street-to-brothel sex workers simply rent a room from the *gharwali* for a fee (“We say ‘this is our client,' we give 20 Rupees and finish”). In this sense, their relationship with the *gharwali* is straightforward (renting a brothel room per client in exchange of a fixed fee) and does not obligate them in any way. Some women say they discuss condom use with clients upon solicitation in the public place (“If we do not tell them [in the public place], then they come here [to the brothel] and refuse. Should we send them back? There only we finish the discussion”). Other women do not discuss condom use in public places; instead “we say yes in front of him… then when he is enjoying, we remove it [condom] from the packet and tell… When his thing is up then he has to do… so he uses.”

#### 3.3.2. Street-to-Lodge

Street-to-lodge sex workers prefer to take clients to familiar lodges, to protect themselves from possible problems from clients (“If we go to a lodge where we do not know anything or anyone, what to do if they do something?”). In Karnataka, these sex workers were shown to have high HIV and STI prevalence, and a relatively high client volume [8]. Factors associated with lodge-based sex work also apply to street-to-lodge sex work: lack of time to negotiate condom use (“10 to 15 minutes. You [have to] do, finish and just come out”), limited number of lodges where they can work (“We have to listen to him so that next time he will let us in, so we have to manage him carefully”), and fear of police raids (“There is fear from the police, they are the problem”). 

#### 3.3.3. Street-to-Street

Street-to-street sex workers take most clients to spaces behind buildings, fields or other public areas where they can have privacy. They use public venues for sex because clients cannot afford room rent, are afraid of police raids in lodges, or feel they have no alternative (“I am afraid of the police. And one or the other lodge will be shut. Clients used to tell [to go to open places instead of lodges]… I used to say ‘I won't come outside,' but they used to say ‘Why spend on lodge and take us there?'”). Street-to-street sex workers need not deal with network operators but are particularly vulnerable to harassment and violence from clients (“He took me to the graveyard… There were 5-6 people totally drunk. They did like that only and did not even give me a rupee. They did without condom”). 

#### 3.3.4. Street-to-Rented-Room

Sex workers sometimes take clients to rooms in houses located in “regular” neighbourhoods; the wider community is usually unaware that sex work happens there (“There will be no fear of police, it will be like a family house”). The understanding between the sex workers and the owners of the house (usually a former sex worker) is straightforward; the women pay a fixed amount per client. While some street-to-rented-room sex workers discuss condom use with the client after arriving at the room, many prefer to clarify the matter upon solicitation to make sure that the client will be using condom (“As soon as we find him we tell him about condom use.”).

#### 3.3.5. Street-to-Home

If their family situation permits it, some sex workers take clients from public places to their own homes; this location is preferred because they “feel safe” there and it saves them money, although they are sometimes bothered by neighbours who “talk and all”. Because of this concern, some women negotiate condoms with clients as soon as they pick them up in public places (“When they are picking us up we tell them ‘If you are going to use condom then I will come, otherwise I won't come”). However, others prefer to discuss condom use after they arrive at their homes (“When they come only we tell them ‘You should use, otherwise go away.'”) Compared to other street-based sex workers, they are less likely to visit drop-in-centres. 

### 3.4. Home

We distinguish between two main types of home-based sex workers (*Devadasis* and non-*Devadasis*), as this distinction has important implications on their sex work practice and associated vulnerabilities. *Devadasis* are women who have been dedicated to a goddess and who, following this initiation, are socially sanctioned to engage in sex work; this tradition (particularly prevalent in Northern Karnataka) has been documented elsewhere [17]. The local *Devadasis* are well known in their communities, particularly in villages. Therefore, clients know their location and come to their homes directly (“When they tie beads [the initiation into the *Devadasi* tradition includes a ritual of tying beads], then people… they come to know, so they come”). 

Other women (not related to the *Devadasi* tradition) also practice sex work from their own homes. They usually start their careers by soliciting clients in other settings for example, public places. Having developed a clientele, they then practice sex work from their homes; their regular clients sometimes act as informal nonremunerated intermediaries with other potential clients. With the exception of clients and sometimes auto-rickshaw drivers, the women work autonomously. 

While *Devadasis* usually have the support of their families and neighbours (“The village people came and stood by me [saying] ‘We have only kept her, she is poor… She is taking care of the whole family'”), non-*Devadasis* are vulnerable to harassment from neighbours. Regarding condom use, for *Devadasis,* family members may provide support if the client refuses to use a condom (“I do [sex work] at home and there are people all around. He [the client] also feels that, because he knows that if he shouts or misbehaves he will be taken to task. So if he wants to use [condom] he will use and do, otherwise he will not [have sex], he will go”). Other home-based sex workers do not have the same support system in place. 

Because non-*Devadasis* try to keep their activities secret, they can be difficult to identify and incorporate in prevention activities. Home-based sex workers are less likely to visit the drop-in-centres and programme clinics (“It's not my habit to go. I am at home only”). 

### 3.5. Phone Network

Phone-based sex workers solicit most clients through the phone (“I get clients only on the phone, I don't go anywhere”). The women work as a network, in that former/more established sex workers get clients for newer women, a service for which they charge a fee. The place of sex is the house of the sex worker acting as intermediary, a lodge or someone else's house. The women start by getting clients through other sex workers but after a while practice sex work independently and deal with clients directly (“I started giving my number to the customers and they started calling me”). The transition between the two stages is described as smooth and unproblematic. 

If the client is attained through another sex worker, the woman has limited ability to refuse the client, even if he becomes violent (“They say ‘Take like this, take like that, take in the mouth.' I said no and he was drunk and started shouting… Aunty [the woman who mediated the meeting] said don't take such things seriously… the aunty was there with me”). If the client is entertained in a lodge, the risks pertaining to lodges mentioned earlier apply (“I don't like to do in the lodge, if it's a house then it's better… There is no fear of police”). These women report experiencing harassment/violence from clients (“Clients are also very bad”; see the above quote). While phone-based sex workers have a wide range of rates, many women charge high fees and their clients are “‘hi-fi' people: businessmen and such.” Sex workers and their clients may consume alcohol before sex (“Those who come for the night drink”). 

The women claim they use condoms consistently with all their clients (“All [clients] use [condoms], nothing like that; they are also concerned about their body”). However, one participant who is new to sex work eventually admitted that at times she has sex without using condom (“If a time comes like that, then we may do [without condom] just for once and come back…”).

In Belgaum, efforts are made to identify and train peer educators who are part of phone-based sex work networks, so that they promote safe sex among this group. However, in most places phone-based sex workers are not covered by HIV programmes, as they are difficult to identify and follow up. Given their concern for anonymity, the women are reluctant to visit drop-in-centres and clinics regularly. 

### 3.6. Parlour

While much of the mediation between sex workers and clients is through the phone, parlour girls differ from phone-based sex workers in that they use their work to solicit clients (“We go to do facials to doctors or their wives or at weddings, then they tell”). Another study participant also solicits clients through “certain women who come to my parlour.” Some of this mediation is done free of charge, while other women who provide clients charge commission (“we give Rupees 500 commission”). Following direct contact at a wedding/private house or referral from a mediator, the woman and the client decide (over the phone) on a place and time to meet, usually a public place for example, restaurant. If they agree on a fee, they go to a hotel or house within or outside the city and have sex there (“They tell it cannot be done at home, so they take us to some other place outside Belgaum or any place where they have a house or something; not here, because it affects the customers”). While the client solicitation is done through the parlour, the sex always takes place outside the parlour, at a place agreeable to the client (“However big the parlour may be, that [sex in the parlour] does not happen, because 12 hours of the day women will be there”). Parlour girls are extremely concerned with maintaining their anonymity (“only those whose houses we visit for facial and all, they know… No one else comes to know”). 

Unlike other sex workers, parlour girls have another source of income; this allows them to have more control over the number and type of clients (“If I am busy and I have customers in the parlour then I refuse”). The clients are usually middle-class men, educated and with well-paid occupations. The women may or may not drink alcoholic beverages while entertaining clients, but clients usually drink (“Yes [they drink], but not much and not hot drinks, just beer, like that”). Nevertheless, the women claim that they can use condoms consistently with their clients (*Interviewer:* “He will use condom?” *Respondent*: “Yes… they are all good clients.”)

To date, little effort has been made to reach these sex workers by programmes, either in Belgaum or elsewhere in Karnataka. This is partly because they are difficult to identify and are presumed to be at lower risk for HIV (because they have a low client volume and a higher level of education compared to that of women practicing sex work in other settings). 

### 3.7. Dhaba

Some women solicit and entertain clients in/around *dhabas* (restaurants located on highways). They do not solicit openly, in front of the *dhaba* (“they do not let us sit in the front”); potential clients talk to the *dhaba* owner or the watchman (“There is a room, the owner sends the clients [there]”). The clients are entertained in rooms or in the fields (“During the day we do [sex] in the room and in the night we go in the fields”). The owner facilitates the transaction with the clients and provides a place for sex, for which he charges approximately a third of the total price (“[he gets] 50, and we keep 100”). When the sex takes place in the fields nearby the *dhaba*, the watchman looks out for the women and charges a fee for this service (“He stands guard for us until we finish, at a distance, then brings us back. What if someone does something?”). Because most clients of *dhaba*-based sex workers are truck drivers who work on specific routes, there is usually an expectation of a significant turnover of women (“They ask for someone new and when they [owners] say ‘no one else is there,' they go away”); hence, many women work in several *dhabas* at the same time (“I go to other *dhabas* also”). Although *dhabas* are somewhat organized sex work settings, sex workers are fairly autonomous and can change the place of work if/when they wish. 

*Dhaba*-based sex workers have a high client volume (“above 10 clients”) and their ability to decline clients and insist on condom use largely depends on the owner's attitude. Study participants reported good relations with the owners and a positive attitude towards safe sex. Police raids represent an important concern for *dhaba*-based sex workers. However, they often feel better protected from the police than those working in lodges, mainly because the location of *dhabas* offers them better prospects of flight and escape (“There is a field at the back and grass is piled, we hide there… The moment the vehicle comes he [owner] warns us to go”). Most clients are “truck drivers,” known for their risky sexual behaviour, in terms of number and type of sexual partners and type of sex preferred (“Back side… and in the mouth… It's like that now, at present that's the trend”) [18]. There is high alcohol consumption among both clients and sex workers (“If you do not drink, you cannot do at all… How many you do, that you do not keep count when you are drunk”). 

Due to their location on highways, it is difficult for programme staff to provide free condoms in *dhabas*. Moreover, *dhaba*-based sex workers are difficult to identify and follow up. They are disinclined to visit drop-in-centres and clinics regularly, and consequently represent one of the poorest covered high-risk groups of sex workers. 

### 3.8. Highway

Highway-based sex work is a form of out-of-town street-to-street sex work. The women solicit clients by standing on the side of the highway and signaling to vehicles. They entertain clients there and then or get into the vehicle and travel for a while, after which they get down and pick up another vehicle (“After the client is done, we catch a different truck”). They have certain routes that they use according to convenience and client demand. Regarding the place of sex, “some do in the vehicle only and some get down in the fields and do”. Highway-based sex workers work without network operators; however, they tend to travel together and/or look out for each other (“My friend will be there, if I go alone I will feel scared. I wait until she finishes and she also waits for me”). 

Highway-based sex workers tend to be at high risk. They have high client volume (around “9-10” clients daily). However, some clients pick up a woman and spend 1-2 days with her. Because they practice sex work independently, highway-based sex workers have freedom to choose their clients. They are less concerned with police raids and harassment from *gundas* (“I have never been caught”). However, they are particularly vulnerable to harassment/violence from clients, who can take advantage of being alone with the women (“[If] I try to tell him and he still refuses to use, we do like that [without condom] only and come. What to do if he won't use at all?”). Most clients are “truck drivers” and tend to “drink” when visiting sex workers. 

Because they work on highways, it is difficult for the programme to identify and follow up women (“Peers don't come here [on the highway]”), and they tend not to visit drop-in-centres (I come only to the clinic… We go on trucks so I can't come”). Hence, they represent another high-risk sex worker group not well covered by the programme. 

### 3.9. Agriculture/Construction Workers

Some women solicit clients at/through their places of work, which are in nonsex work related industries (e.g., agriculture, construction). The interviews conducted were with agricultural workers (one participant also did construction work). 

The women solicited/entertained clients before or after work (“While coming from work I do or while going. I don't go to do that only”). Once a woman had agreed to paid sex, other village men find out and approach the woman (“I found him first and then he told others”). Mediation is also done through other agricultural workers who practice sex work; they support each other (“They stay and wait for us. We go together after we are finished”). These arrangements are informal and free of charge. After the woman establishes contact with the client, they decide on the place and time of the meeting: sex usually takes place in “agricultural fields”. 

Agricultural labourers practice sex work without external pressure from their employers (“if she wants she can do, if she does not she need not do”). Anecdotal evidence suggests that some contractors harass female construction workers into having sex with them (“I have done with the construction workers. Otherwise they wouldn't take me for work nor give me my wages”); however not enough data are available to clarify this issue. 

While agricultural labourers have few clients, they are at risk of HIV through a combination of factors. They can be difficult to identify and follow up, and are unlikely to visit drop-in-centres and clinics usually located in nearby towns (“Women in the villages… They do not know much about condom… They cannot spend [money on] bus charge and come [to the clinic]. And who will go and tell them that clinic will be held?”). This may affect women's levels of knowledge about HIV (e.g., one participant in contact with the programme for one year did not know what HIV is). Because of the lack of programmes and pharmacies in villages, condom availability is often problematic. 

## 4. Discussion

We have described the mode of operation of the Belgaum sex work industry, in its many forms, and vulnerability factors specific to each setting (summary provided in Table 1). In addition to the previously documented brothel-based, lodge-based, street-based, *dhaba*-based, and highway-based sex workers, the emergence of other sex work categories in Karnataka should be noted, including phone-network, parlour, and agricultural workers. Women practicing sex work in brothels, lodges, *dhabas,* and on highways are at high-risk for HIV. Of these, *dhaba* and highway-based sex workers are the least covered by HIV intervention programmes. 

This study identified thirteen different settings where women practice sex work in Belgaum and represents the most finely-tuned description of sex work to date [19]. Understanding the wide range of sex work settings and their *modus operandi* is important to HIV programming, as it allows the development of appropriately tailored intervention programmes. While in India this issue has mostly been examined quantitatively [8, 20], our study employs qualitative data. 

Quantitative biobehavioural survey data have shown that brothel and street-to-lodge sex workers are at highest risk for HIV in Karnataka [8]. As documented in other regions in India [5, 21–23], much of the risk associated with working in brothels, lodges, and *dhabas* appears due to the presence and role of the network operators, who control the type and number of clients and the extent to which the women can use condoms and be reached by programmes. As observed throughout the world [24], highway-based women are at high HIV risk because they work in isolated places, leaving them vulnerable to possible harassment/violence from clients. In India, their clients are mostly truck drivers, known for their risky sexual behaviour (e.g., many sexual partners, preference for anal sex) [18]. Their isolation also makes them more difficult to identify and follow up by programme staff.

This is one of the first papers to describe the modes of operation of emerging sex work settings in Karnataka, namely, phone-based, parlour girls, and agricultural workers. These sex work types, especially phone-based, are likely to increase greatly in the future. Phones are also increasingly used by women working in other settings, as also documented in other South Indian states [20]. This may result in an “invisible” sex work industry parallel to the “visible” sex work industry; HIV programmers need to keep up with these changes to develop appropriate outreach strategies. 

Previous research has shown a variation in HIV risk between different sex worker categories in Karnataka [4, 8, 20]. However, the places of solicitation and sex do not *in and of themselves* determine degree of risk; they are often proxies for other vulnerability factors specific to each sex work setting. For instance, condom use is shown to be the result of a complex decision-making process influenced by a multitude of factors, such as the rate charged per client, the woman's level of autonomy, the presence and role of network operators, the amount of time spent with the client, the experience of violence and forced sex, the sociodemographic and economic profile of the client, the woman and client's alcohol consumption, the place of condom negotiation, the level of exposure to HIV prevention programmes and access of their services, the availability of condoms, and the woman's condom negotiation skills. 

The study only involved interviews with sex workers. Reports from sex workers' clients, network operators, and programme staff could have been used to complement the women's discourse. Other studies have examined the perspectives of some of these actors. For example, Aubé-Maurice conducted a qualitative study on the role of gender on HIV risk among the clients of sex workers (working in their homes, public places, and brothels) from Belgaum district and found that clients perceive sexual relations with sex workers as a vice, but also as an opportunity to conform to the masculine ideal to sexually satisfy women [25]. A study that examined reports of *gharwalis* from Nagpur, Maharashtra documented the feasibility of involving *gharwalis* in brothel-based HIV prevention programs [22]. 

The data consist of 50 interviews with sex workers; an average of 4 interviews were conducted per sex work setting, hence data saturation may not have been achieved. Study participants were selected with the help of peer educators working with BIRDS. While this helped the enrollment process, sex workers who are in contact with peer educators are likely to be sex worker collective members and to have access to the services offered by the HIV programme. Alcohol consumption may be an important vulnerability factor [13–15]. However, although specifically asked about the women's and their clients' alcohol consumption and its effect on condom use, in many sex work settings women were reluctant to admit that they drink alcohol beverages, possibly due to the strong stigma against alcohol consumption in India, especially among women. 

Previous studies have documented interdistrict variations in the way sex work operates in Karnataka [23]. The results cannot therefore be extrapolated to the entire state/region. However, based on our extensive work throughout the state, we believe that most sex work settings documented in Belgaum can also be found in other Karnataka districts. Similar studies on this topic in other parts of Karnataka and elsewhere will contribute to increasing the understanding of the sex work industry in India. 

Future quantitative surveys of sex workers may need to incorporate more subtle and elaborate measures of the sex work typology, including the place(s) of client solicitation, the place(s) of sex, extent of phone use for soliciting, and the presence and role of network operators. This approach is increasingly supported by other researchers in India [20]. When conducting mapping of sex workers and deciding the sampling method, researchers deploying quantitative surveys need to take account of the heterogeneity of sex work settings within a given area. Failure to do so may result in underrepresentation of certain sex worker types, often the “invisible” and/or hard-to-reach sex workers. HIV programmes currently decide whom to target largely on the basis of quantitative data; hence, lack of data on certain sex work categories (e.g., *dhaba*, highway) can result in poor programmatic coverage of potentially high-risk groups of sex workers. 

Given the variety of sex work settings and the variation in the level of sex workers' exposure to the HIV programme, outreach strategies specific to each sex work setting may need to be developed. For example, while drop-in-centre and peer educator models seem to be ensuring adequate coverage of street-based sex workers, they are less useful for identifying and following up *dhaba* and highway-based sex workers. Similarly, phone-based sex workers and parlour girls can be reached using outreach strategies that use the networks employed by the women to practice sex work. Involvement of network operators is also important to provide comprehensive services to brothel and lodge-based sex workers, as they play an important role in the women's ability to practice safe sex. An attempt should be made to identify agricultural workers that practice sex work and offer them services in their home villages/localities, given their isolation from the towns where most drop-in-centers and clinics are located. 

The paper presents a comprehensive description of the mode of operation of the female sex work industry in Belgaum district, Karnataka and the HIV risk and vulnerability factors related to each sex work setting. The factors associated with vulnerability are often interrelated and play out in different ways in different contexts. The approach of collectivization and provision of services through stand-alone sites and outreach may not be sufficient to provide access to services for the more hidden networks of women. Novel approaches for providing services for these women need to be explored. 

## Figures and Tables

**Table 1:** Summary of main vulnerability factors by type of sex work setting.

Sex work setting	Main vulnerability factors
Brothel	(i) High client volume.
	(ii) Autonomy limited by the *gharwalis*.
	(iii) Harassment and violence from the *gharwalis*.
	(iv) High alcohol consumption.
	(v) Their contact with the HIV prevention programme staff depends on the *gharwali*.

Lodge	(i) High client volume.
	(ii) Autonomy limited by the lodge manager.
	(iii) Limited number of lodges so pressure to conform to lodge rules.
	(iv) Police raids.
	(v) Time with the client controlled by lodge.
	(vi) Need to buy condoms from the lodge manager.
	(vii) Their contact with the programme staff depends on the lodge manager, who runs the lodge like a business.

	(i) Autonomy to choose clients limited by the competition between sex workers and the number of available clients.
	(ii) Harassment and violence from *gundas* and police members.
	Vulnerability factors specific to the place of sex
	Street-to-lodge FSWs:
	(i) Medium to high client volume.
	(ii) Autonomy limited by the lodge manager.
Street	(iii) Limited number of lodges so pressure to conform to lodge rules.
	(iv) Time with the client controlled by lodge.
	(v) Police raids.
	Street-to-street FSWs:
	(i) Harassment and violence from clients.
	Street-to-home FSWs:
	(i) Harassment from neighbours.

Home	(i) Non-*Devadasis:* harassment from neighbours.
	(ii) Non-*Devadasis:* difficult to identify by the programme.

Phone network	(i) Police raids if entertaining clients in lodges.
	(ii) Difficult to identify and stay in contact with the programme.

Parlour girls	(i) High expectation from clients.
	(ii) Difficult to identify and stay in contact with the programme.

*Dhaba *	(i) High client volume.
	(ii) Autonomy limited by the *dhaba* manager.
	(iii) Most clients are truck drivers.
	(iv) High alcohol consumption among clients and FSWs.
	(v) Difficult to identify and stay in contact with the programme.

Highway	(i) High client volume.
	(ii) Harassment and violence from clients.
	(iii) Most clients are truck drivers.
	(iv) High alcohol consumption among clients.
	(v) Difficult to identify and stay in contact with the programme.

Agricultural workers	(i) Little knowledge about HIV and safe sex.
	(ii) Lack of condom availability in villages.
	(iii) Difficult to identify and stay in contact with the programme.
